# Cigarette smoke‐induced HMGB1 translocation and release contribute to migration and NF‐κB activation through inducing autophagy in lung macrophages

**DOI:** 10.1111/jcmm.14789

**Published:** 2019-11-26

**Authors:** Yanqing Le, Yanhong Wang, Lu Zhou, Jing Xiong, Jieyu Tian, Xia Yang, Xiaoyan Gai, Yongchang Sun

**Affiliations:** ^1^ Department of Respiratory and Critical Care Medicine Peking University Third Hospital Beijing China; ^2^ Department of Respiratory Medicine Zhongshan City People's Hospital Zhongshan China; ^3^ Hematology Oncology Center Beijing Children's Hospital Capital Medical University Beijing China; ^4^ Department of Respiratory Medicine Tianjin Medical University General Hospital Tianjing China

**Keywords:** autophagy, chronic obstructive pulmonary disease, high‐mobility group box 1, lung macrophage

## Abstract

High‐mobility group box 1 (HMGB1) shows pro‐inflammatory activity in various inflammatory diseases and has been found up‐regulated in chronic obstructive pulmonary disease (COPD). Lung macrophages play an important role in airway inflammation and lung destruction in COPD, yet whether HMGB1 is involved in cigarette smoke (CS)‐induced lung macrophage dysfunction is unknown. We sought to evaluate the intracellular localization and release of HMGB1 in lung macrophages from COPD patients and CS‐exposed mice, and to investigate the role of HMGB1 in regulating autophagy in CS extract (CSE)‐treated lung macrophages (MH‐S cells). Our results showed that HMGB1 was highly expressed in lung tissues and sera of COPD patients and CS‐exposed mice, along with predominantly cytoplasmic exporting from nuclei in lung macrophages. In vitro experiments revealed that CSE promoted the expression, nucleocytoplasmic translocation and release of HMGB1 partly via the nicotinic acetylcholine receptor (nAChR). Blockade of HMGB1 with chicken anti‐HMGB1 polyclonal antibody (anti‐HMGB1) or glycyrrhizin (Gly) attenuated the increase of LC3B‐II and Beclin1, migration and p65 phosphorylation, suggesting the involvement of HMGB1 in autophagy, migration and NF‐κB activation of lung macrophages. Hydroxychloroquine (CQ), an autophagy inhibitor, enhanced the increase of LC3B‐II but not Beclin1 in CSE or rHMGB1‐treated MH‐S cells, and inhibition of autophagy by CQ and 3‐methyladenine (3‐MA) abrogated the migration and p65 phosphorylation of CSE‐treated cells. These results indicate that CS‐induced HMGB1 translocation and release contribute to migration and NF‐κB activation through inducing autophagy in lung macrophages, providing novel evidence for HMGB1 as a potential target of intervention in COPD.

## 1 INTRODUCTION

Chronic obstructive pulmonary disease (COPD) is characterized by persistent airway inflammation and lung destruction, with increased numbers of lung macrophages, neutrophils and lymphocytes in airways, lung parenchyma and pulmonary vessels.^1^ Lung macrophages play a critical role in clearing inflammatory mediators and maintaining of lung homeostasis and are key mediators in the pathogenesis of COPD.^2^, ^3^

In COPD, lung macrophages are increased but with impaired functions, which may responsible for pathogenesis of the disease.^3^, ^4^ A growing body of evidence indicates that autophagy plays a critical role in regulating production, recruitment, polarization and phagocytosis of macrophages,^5^, ^6^ and findings from patients and animal models showed activation of autophagy in the lungs of COPD.^7^, ^8^ Autophagy is a lysosomal degradation pathway and a physiological process associated with clearance of aggregated proteins and damaged organelles, which are critical for maintenance of cellular homeostasis.^9^ Recent studies showed that cigarette smoke (CS) induced autophagy activation in macrophages.^10^, ^11^ However, the underlying molecular mechanisms have not been fully elucidated.

High‐mobility group box 1 (HMGB1) is a DNA‐binding nuclear protein, contributing to stabilization of chromatin structures and modulation of target gene transcription.^12^ HMGB1 is present in the nuclei of almost all eukaryotic cells, especially highly expressed in macrophages, epithelial and endothelial cells, and has been implicated in several lung diseases.^12^, ^13^ Under endotoxin or endogenous pro‐inflammatory cytokine conditions, HMGB1 can be transported from the nucleus to the cytoplasm, a process called nucleocytoplasmic translocation, and then released from the cell to the extracellular milieu.^12^, ^14^ Extracellular HMGB1, as a danger‐associated molecular pattern (DAMP), has been shown to be involved in a variety of inflammatory diseases,^15^ such as sepsis,^16^ asthma 17 and arthritis.^15^, ^18^ Evidence from COPD patients revealed high expression of HMGB1 in lung tissue, bronchoalveolar lavage fluid (BALF), sputum and plasma, and it was negatively correlated to post‐bronchodilator FEV1/FVC ratio of lung function.^19^, ^20^ In acute CS‐exposed mouse models, HMGB1 translocation and release in lung epithelial cells mediated lung inflammation.^21^ Collectively, these findings implicate the involvement of HMGB1 in the pathogenesis of COPD, but its exact role is not clear. Because studies have illustrated that released HMGB1 exerts pro‐inflammatory effects through the induction of autophagy in macrophages,^22^, ^23^ we, therefore, hypothesized that CS‐induced HMGB1 translocation and release might participate in pathogenesis of COPD by regulating autophagy of lung microphages.

## 2 MATERIALS AND METHODS

### 2.1 Reagents

Recombinant (r) HMGB1 was purchased from R&D Systems and chicken anti‐HMGB1 polyclonal antibody (anti‐HMGB1) and chicken IgY control were from Shino‐Test Corporation. Glycyrrhizin (Gly), mecamylamine (MEC), hydroxychloroquine (CQ) and 3‐methyladenine (3‐MA), all water‐soluble, were obtained from Sigma‐Aldrich Corporation. Antibodies of HMGB1, CD68, F4/80, LC3B‐I/II and Beclin1 were purchased from Abcam Corporation, while phospho (p)‐p65 (Ser536), p65 and β‐actin were purchased from Cell Signaling Technology. Histon H3 was obtained from Abclonal Biotech. Horseradish peroxidase (HRP)‐conjugated donkey anti‐rabbit or anti‐mouse IgG antibodies were purchased from Cell Signaling Technology. Alexa 594‐labelled goat anti‐rabbit IgG (H + L) and alexa 488‐labelled goat anti‐mouse IgG (H + L) antibody were from Jackson ImmunoResearch while alexa 488‐labelled goat anti‐rat or anti‐rabbit IgG (H + L) antibodies were from Abcam Corporation.

### 2.2 Study subjects

Forty‐five subjects were consecutively recruited in Peking University Third Hospital and were divided into three groups: COPD group, Smoker group and Non‐smoker group. Written informed consent was obtained from all subjects, and the Ethics Committee of Peking University Third Hospital approved this study, and the research was carried out according to the World Medical Association Declaration of Helsinki. Patients in COPD group were subjects with a smoking history of ≥20 pack‐years and post‐bronchodilator FEV1/FVC < 70%, according to the Global Initiative for Chronic Obstructive Lung Disease (GOLD) guidelines.^1^ Subjects with a smoking history of ≥20 pack‐years and normal lung function (post‐bronchodilator FEV1/FVC ≥ 70%) were categorized as the Smoker group. Subjects in Non‐smoker group denied smoking history and showed normal lung function (post‐bronchodilator FEV1/FVC ≥ 70%). Patients with COPD were clinically stable and had not experienced any exacerbations for ≥3 months preceding inclusion in the study. All patients had received surgery for solitary lung nodules, and lung tissues for this study were collected at maximum distance from the pulmonary lesions and without signs of retro‐obstructive pneumonia or tumour invasion.

### 2.3 Cigarette smoke exposure protocol for the mouse model

Procedures for establishment of the mouse model followed the committee's animal care and use guidelines and were approved by the Ethics Committee of Peking University Third Hospital. CS‐exposure protocols were described in our previous study.^24^ Briefly, 6‐8 week old, specific‐pathogen‐free (SPF), female C57BL/6 mice were purchased from Beijing Vital River Laboratory. Mice were exposed to CS (Baisha cigarettes with filter, Hunan, China; tar 11 mg, nicotine 0.9 mg, CO 12 mg) or air by using a nose‐only, directed flow inhalation and smoke exposure system (SG‐300; SIBATA) for 50 min each time, two times a day with 20‐minute free interval, 5 days a week for 24 weeks.

### 2.4 Quantification of emphysema

The mean linear intercept (MLI) and destructive index (DI) were used to quantify emphysema, as described in our previous study.^24^ Briefly, MLI was measured by using a 100 × 100 µm grid passing randomly through the lung. MLI was calculated as the total length of each line of the grid divided by the number of alveolar intercepts. The measurement of DI was performed by a grid with 42 points which were at the centre of hairline crosses superimposed on the lung field. Structures lying under these points were marked as normal (N) or destroyed (D) alveolar and/or duct spaces. Points falling over other structures, such as duct walls, alveolar walls, did not enter into the calculations. DI was calculated as D/ (D + N) ×100%.

### 2.5 Immunohistochemistry and immunofluorescence of lung tissues

Lung tissue sections (5 μm thick) were deparaffinized and treated with 3% H_2_O_2_–CH_3_OH for 15 minutes to block endogenous peroxidase. Samples were submerged in citrate buffer (pH 6.0) in a microwave oven for antigen retrieval, blocked with 5% BSA for 30 minutes at room temperature and then incubated overnight with antibody HMGB1 (1:1000). After washing with PBS, slices were incubated with horseradish peroxidase (HRP)‐conjugated goat anti‐rabbit IgG (ZSGB‐Bio) at 37°C for 30 minutes and then stained with DAB detection system kit (ZSGB‐Bio). HMGB1 expression and localization in the lung were detected under light microscopy and analysed by image‐pro plus 6.0 software.

For immunofluorescence, lung tissue sections were incubated overnight with antibodies HMGB1 (1:600), CD68 (1:100) or F4/80 (1:100). After washing with PBS, sections were incubated with alexa 594‐labelled goat anti‐rabbit secondary antibody and alexa 488‐labelled goat anti‐mouse/rat secondary antibody for 1 hour at 37°C, and then 4, 6‐diamidino‐2‐phenylind‐ole dihydrochloride (DAPI) (Beyotime) were added for cellular nuclear staining. Co‐localization of HMGB1 and CD68 or F4/80 was evaluated with confocal microscopy (TCS‐SP8; Leica Microsystems).

### 2.6 Measurement of HMGB1 in serum and supernatants

Serum samples and cell culture supernatants were collected and used for assessing HMGB1 level by enzyme‐linked immunosorbent assay (ELISA), according to the manufacturer's instructions for human HMGB1 ELISA kit (Elabscience) and mouse HMGB1 ELISA kit (Elabscience).

### 2.7 Cell culture and treatment

The MH‐S cells, a mouse alveolar macrophage cell line, were purchased from Bio‐Rad Biological Technology Co. Ltd, an agent of ATCC. Cells were cultured in RPMI‐1640 medium (Hyclone) containing 10% foetal calf serum (Gibco) at 37°C and 5% CO_2_. Medium was changed every day, and cells were seeded to proper culture plates at a density of 10^5^cells/cm^2^ for the following experiments. Different concentrations of CS extract (CSE) or rHMGB1 were then added to the culture medium for indicated time with or without pre‐treatment by anti‐HMGB1, Gly, MEC, CQ and 3‐MA.

### 2.8 CSE preparation

CS extract was prepared as described in our previous study.^24^ In brief, smoke fogs from five cigarettes were passed through 10 mL of RPMI‐1640 medium at a constant airflow and then was sterile filtered through a 0.22 µm filter. Absorbance value was constant (4.0 ± 0.05) by measuring at a wavelength of 320 nm.

### 2.9 Cell viability by MTT assay

Cell viability was tested with the MTT assay. Cells were seeded into 96‐well plates with 2000 cells/well. Cell viability was assessed using the MTT proliferation assay kit (Applygen) according to the manufacturer's instructions. Absorbance was read in a spectrophotometer at a wavelength of 570 nm.

### 2.10 Immunofluorescence and immunocytochemistry of cultured cells

Immunofluorescence and immunocytochemistry were performed to evaluate the protein localization of HMGB1. Cells were grown on glass bottom cell culture dishes (Nest, Wuxi, China). The treated cells were then fixed with 4% paraformaldehyde for 10 minutes, incubated with 0.2% Triton X‐100 for 10 minutes, blocked with 5% BSA for 30 minutes and then incubated overnight with anti‐HMGB1 antibody (1:600) at 4°C. These dishes were incubated with alexa 488‐labelled goat anti‐rabbit secondary antibody for 1 hour at 37°C, and then, DAPI was added for 10 minutes. Or after the dishes were incubated overnight with anti‐HMGB1 antibody, they were incubated with horseradish peroxidase (HRP)‐conjugated goat anti‐rabbit IgG for 30 minutes at 37°C and then stained with DAB detection system kit.

### 2.11 Nuclear and cytoplasmic extraction

To evaluate the protein localization of HMGB1, we extracted cellular nuclear protein and cytosolic protein by using NE‐PER™ Nuclear and Cytoplasmic Extraction Reagents (Thermo Fisher Scientific), and the HMGB1 expression in nuclear or cytoplasmic protein extracts was detected by Western blot. β‐actin and Histon H3 were used as internal reference proteins for the cytoplasmic and nuclear fractions, respectively.

### 2.12 Concentration and purification of culture supernatants

The supernatants of treated MH‐S cells were added to 10 KDa centrifugal filter units from Millipore Company for concentration and purification following the manufacturer's instructions. Western blot was used to detect extracellular HMGB1 level, and the volume of loading was determined by the number of cells.

### 2.13 Western blot for protein expression

To evaluate the protein expression of HMGB1, LC3B‐I/II, Beclin1, p‐p65 and p65, cellular protein extracts were subjected to 10% or 12% SDS‐PAGE and transferred to 0.22 µm PVDF membrane (Millipore), and the membranes were incubated overnight with antibodies as follows: HMGB1 (1:1000), LC3B‐I/II (1:2000), Beclin1 (1:2000), p‐p65 (1:1000) and p65 (1:1000). These membranes were incubated with HRP‐conjugated donkey anti‐rabbit or anti‐mouse IgG antibody (1:1000, CST) as the secondary antibody for 1 hour at room temperature and then visualized by enhanced chemiluminescence (Millipore).

### 2.14 Migration assay

Cells were seeded in the upper chamber of transwell plates (24‐well, 8.0 μm pore membranes) (Corning). After treated and incubated for 24 hours at 37°C, the cells remaining at the upper surface of the membrane were removed with cotton swabs, and the cells on the lower surface of the membrane were the migrated cells. After fixed with 4% paraformaldehyde and stained with 0.1% crystal violet solution, the cells that passed through the filter were photographed by microscopy.

### 2.15 Statistical analysis

All the data were expressed as mean ± standard error of the mean (SEM) and analysed using SPSS 20.0 software. Two‐group comparisons were performed using Student's *t* test. Three or more group comparisons were performed with one‐way analysis of variance (ANOVA) accompanied by Bonferroni post hoc test (equal variances assumed) or Dunnett's T3 (equal variances not assumed) post hoc tests. Values of *P* < .05 were considered to be statistically significant.

## 3 RESULTS

### 3.1 Demographic characteristics of study population

Fifteen patients with COPD, fifteen smokers with normal lung function, and fifteen non‐smokers with normal lung function were recruited. The characteristics of these subjects were summarized in Table 1.

**Table 1:** Demographic characteristics of study population

Parameters	Non‐smoker	Smoker	COPD (GOLD I/II)
Subjects (male, n)	15	15	15
Age (Years)	52.86 ± 14.61	57.38 ± 12.15	65.43 ± 8.34
BMI (kg/m^2^)	24.51 ± 3.24	23.96 ± 2.34	23.36 ± 2.72
FEV1 (%)	95.37 ± 12.79	90.59 ± 16.84	71.34 ± 13.53
Post‐bronchodilator FEV1/FVC (%)	80.70 ± 3.02	80.00 ± 6.52	63.96 ± 5.31
Smoking index (pack‐years)	0	31.40 ± 11.06	41.54 ± 19.19

Note

Smoking index = average number of cigarettes per day (pack) × number of years of smoking history (years); BMI = weight (kilograms, kg)/ in square of height (square of metres, m^2^).

Abbreviations: BMI, body mass index; COPD, chronic obstructive pulmonary disease; FEV1, forced expiratory volume in 1 s; FVC, forced vital capacity; GOLD, Global Initiative for Chronic Obstructive Lung Disease guidelines.

### 3.2 HMGB1 was highly expressed and underwent nucleocytoplasmic translocation in lung macrophages from COPD patients

**Figure 1:**
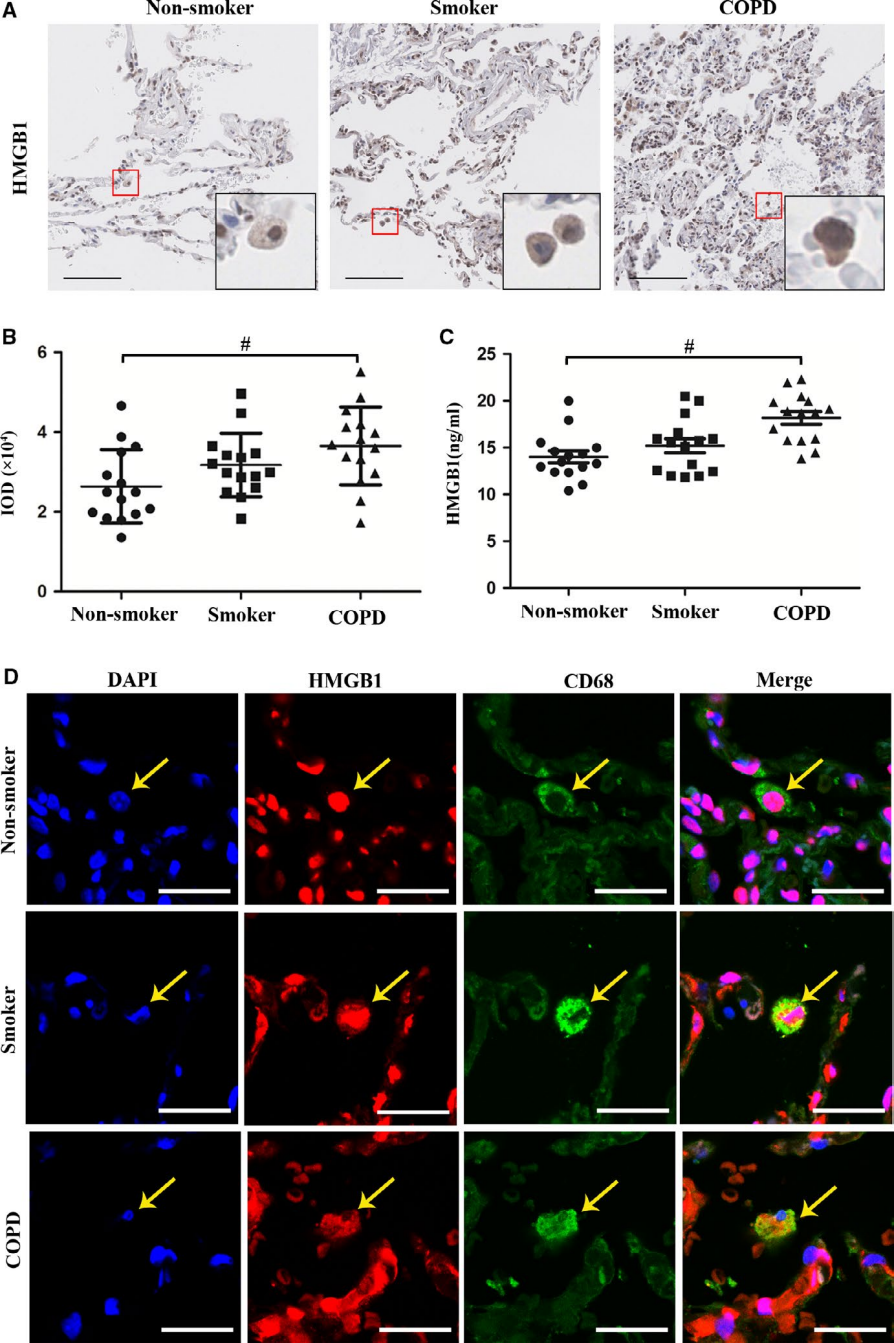
HMGB1 was highly expressed and underwent nucleocytoplasmic translocation in lung macrophages from COPD patients. A, Representative immunohistochemistry of HMGB1 in lung tissues of COPD, Smoker and Non‐smoker groups. Bar: 100 μm. B, The integrated optical density (IOD) in immunohistochemistry of HMGB1. C, Level of HMGB1 was measured in serum. D, Representative immunofluorescence of co‐localization of HMGB1 and CD68 in lung tissue of COPD, Smoker and Non‐smoker groups. Bar: 25 μm. ^#^ *P* < .05. N = 15 in each group. ^#^ *P* < .05. Values are mean ± SEM

Studies showed that HMGB1 was expressed in lung macrophages of COPD patients.^25^ To confirm these findings and to further evaluate the intracellular localization of HMGB1 in COPD patients, smokers and non‐smokers, we performed immunohistochemistry and immunofluorescence in lung tissues from these subjects undergoing lung resection for indicated diseases. HMGB1 expression in lung tissues was significantly increased in COPD group compared with Non‐smoker group (Figure 1A‐B). Changes in serum HMGB1 levels showed a similar trend (Figure 1C), suggesting the release of HMGB1. Furthermore, immunohistochemistry showed that HMGB1 was detected almost only in the nuclei of macrophages in Non‐smoker group, while it was detected both in the cytoplasm and the nuclei of macrophages in COPD and Smoker groups (Figure 1A), indicating that HMGB1 was translocated from the nuclei to the cytoplasm in the latter two groups. Immunofluorescence also showed that HMGB1 had a similar pattern of intracellular localization and was co‐localized with CD68, a marker of human macrophages (Figure 1D). These results indicated that HMGB1 underwent up‐regulation and nucleocytoplasmic translocation in lung macrophages from COPD patients.

### 3.3 HMGB1 was highly expressed and underwent nucleocytoplasmic translocation in lung macrophages from the CS‐induced COPD model

**Figure 2:**
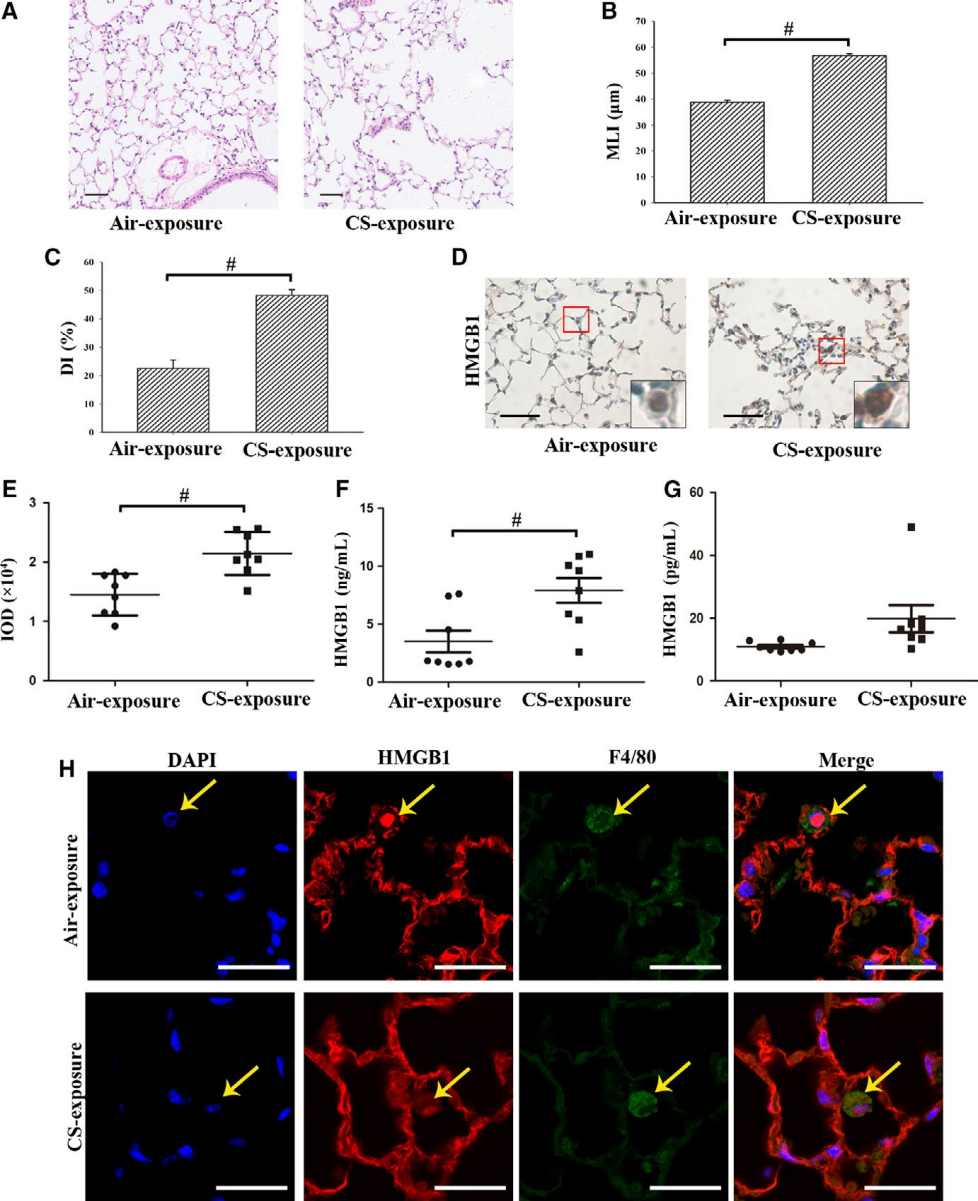
HMGB1 was highly expressed and underwent nucleocytoplasmic translocation in lung macrophages from the CS‐induced mouse model. A, Representative H&E‐stained lung sections showed lung destruction with enlargement of airway spaces. Bar: 50 μm. (B‐C) Quantification of emphysema by mean linear intercept (MLI) and destructive index (DI). D, Representative immunohistochemistry of HMGB1 in lung tissues of CS‐exposed and air‐exposed mice. Bar: 50 μm. E, The integrated optical density (IOD) in immunohistochemistry of HMGB1. F, Level of HMGB1 was measured in serum. G, Level of HMGB1 was measured in bronchoalveolar lavage fluid (BALF). H, Representative immunofluorescence of co‐localization of HMGB1 and F4/80 in lung tissue of CS‐exposed and air‐exposed mice. Bar: 25 μm. ^#^ *P* < .05. N = 8 in each group. Values are mean ± SEM

Mice exposed to CS for 24 weeks showed lung destruction, that is enlargement of airway spaces (Figure 2A), with significantly increased MLI (Figure 2B) and DI (Figure 2C), consistent with changes typical of COPD. Immunohistochemistry revealed higher HMGB1 expression and nucleocytoplasmic translocation in lung tissues in CS‐exposed mice (Figure 2D‐E), and immunofluorescence showed a similar pattern of intracellular localization and co‐localization with F4/80, a marker of mouse macrophages (Figure 2H). Besides, the level of HMGB1 in serum from CS‐exposure group was significantly higher (Figure 2F) as compared to Air‐exposure group, although that in BALF showed no significant difference (Figure 2G).

### 3.4 CSE promoted the expression, nucleocytoplasmic translocation and release of HMGB1 in lung macrophages

**Figure 3:**
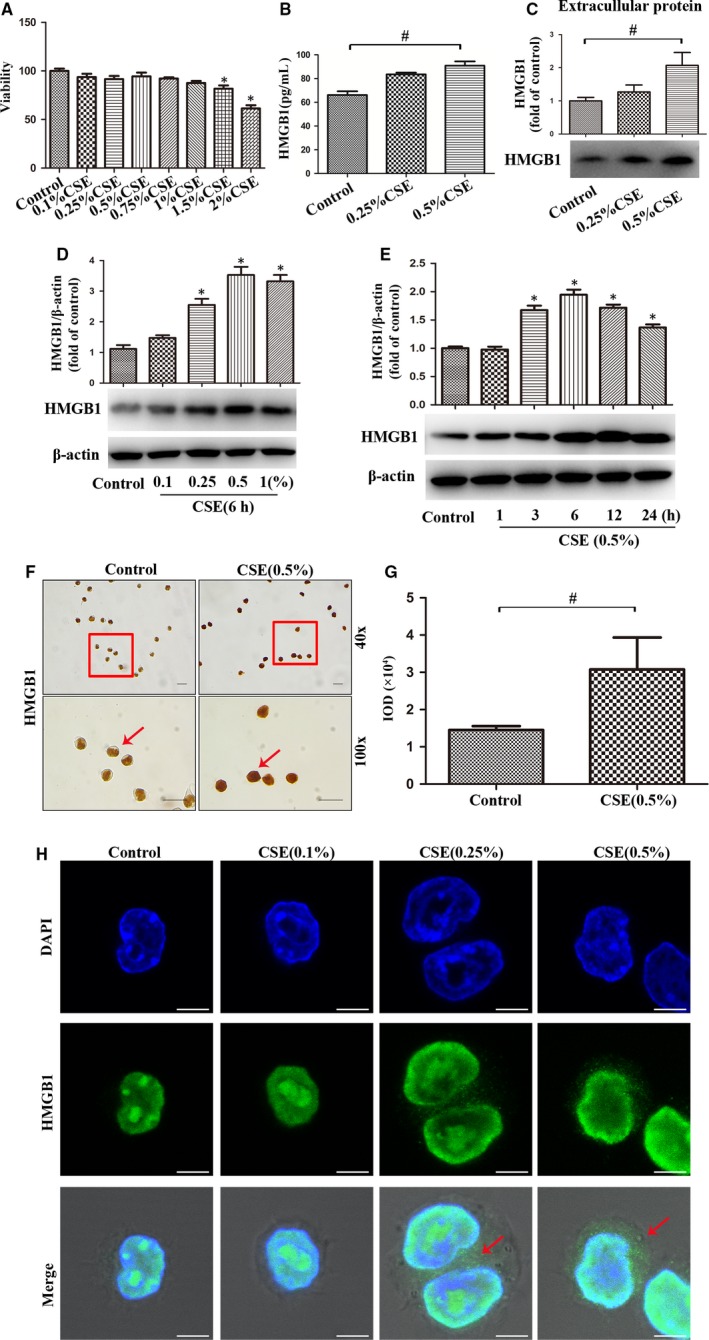
CSE promoted the expression, nucleocytoplasmic translocation and release of HMGB1 in lung macrophages. A, MH‐S cells were treated with CSE for 24 h, and cellular viability was assessed by MTT. (B‐C) After MH‐S cells were treated with CSE (0.25% or 0.5%) for 24 h, level of HMGB1 in cultured supernatants was measured by ELISA and Western blot. D, Expression of HMGB1 was quantified in MH‐S cells after incubation with different concentrations of CSE for 6 h. E, Expressions of HMGB1 was quantified in MH‐S cells after incubation with 0.5% CSE at the indicated time points. F, Expression and localization of HMGB1 in MH‐S cells after CSE incubation for 6 h by immunocytochemistry. Bar: 20 μm. G, The integrated optical density (IOD) in immunocytochemistry of HMGB1. H, Localization of HMGB1 in MH‐S cells after CSE (0.1%, 0.25% or 0.5%) incubation for 6 h was assessed by immunofluorescence. Bar: 5 μm. *P < .05 vs Control group. ^#^ *P* < .05. Values are mean ± SEM of three independent experiments

In order to confirm the in vivo findings of high expression and nucleocytoplasmic translocation of HMGB1 in lung macrophages from COPD patients and CS‐exposed mice, we stimulated MH‐S cells with CSE in an in vitro culture system. Concentrations of CSE less than 1.5% had no effect on cellular viability as determined by MTT (Figure 3A). Therefore, MH‐S cells were stimulated by various concentrations of CSE (0, 0.1%, 0.25%, 0.5% or 1%) for 6 hours or by CSE (0.5%) for indicated time (1, 3, 6, 12, or 24 hours). Western blot results showed that CSE induced concentration‐dependent and time‐dependent HMGB1 expression (Figure 3D‐E). Immunocytochemistry (Figure 3F‐G) and immunofluorescence (Figure 3H) showed that HMGB1 was detected almost only in the nuclei in Control group while it was detected in both the nuclei and the cytoplasm in CSE group, suggesting that HMGB1 was translocated from the nuclei to the cytoplasm in CSE‐induced MH‐S cells. Extracellular HMGB1 was detected in culture supernatants by ELISA (Figure 3B) or in concentrated and purified culture supernatants by Western blot (Figure 3C). Extracellular HMGB1 was significantly increased in CSE group (0.5%) compared to Control group, indicating nucleocytoplasmic translocation and release of HMGB1. These results confirmed that CSE promoted the expression, nucleocytoplasmic translocation and release of HMGB1 in lung macrophages.

### 3.5 Mecamylamine suppressed the expression, nucleocytoplasmic translocation and release of HMGB1 in CSE‐treated lung macrophages

**Figure 4:**
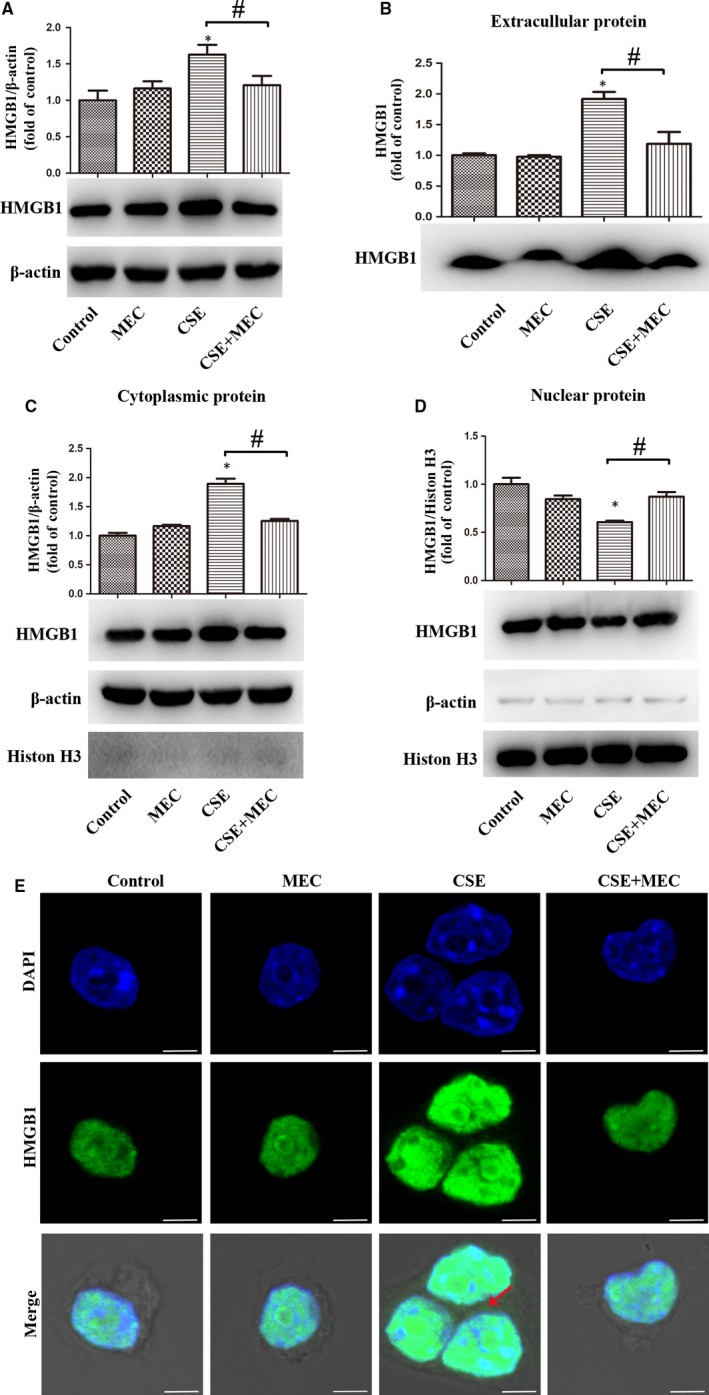
MEC suppressed the expression, nucleocytoplasmic translocation and release of HMGB1 in CSE‐treated lung macrophages. (A‐D) MH‐S cells were pre‐treated with MEC (50 μmol/L) for 90 min before 0.5% CSE incubation for 24 h. HMGB1 was detected in whole protein, extracellular protein, cytoplasmic protein or nuclear protein with Western bolt. E, MH‐S cells were pre‐treated with MEC (50 μmol/L) for 90 min before 0.5% CSE incubation for 6 h. Localization of HMGB1 in MH‐S cells was assessed by immunofluorescence. Bar: 5 μm. *P < .05 vs Control group. ^#^ *P* < .05. Values are mean ± SEM of three independent experiments

In order to clarify whether the effect of CSE was exerted via nicotine and its receptor, we used mecamylamine (MEC) to block the nicotinic acetylcholine receptor (nAChR) in the cell culture system. MH‐S cells were incubated with MEC before CSE stimulation. Western blot was used to detect HMGB1 expression. As expected, CSE‐treated cells had increased HMGB1 in cytoplasmic protein (Figure 4C) and whole protein (Figure 4A), but decreased HMGB1 in nuclear protein (Figure 4D). Notably, MEC reversed these changes in CSE‐stimulated MH‐S cells. Besides, MEC also blocked CSE‐induced extracellular HMGB1 increase (Figure 4B), detected in concentrated and purified culture supernatants by Western blot. Immunofluorescence showed that MEC reversed CSE‐induced HMGB1 nucleocytoplasmic translocation (Figure 4E). Collectively, these data indicated that CSE‐induced expression, nucleocytoplasmic translocation and release of HMGB1 in lung macrophages were dependent on nAChR.

### 3.6 HMGB1 participated in CSE‐induced autophagy of lung macrophages

**Figure 5:**
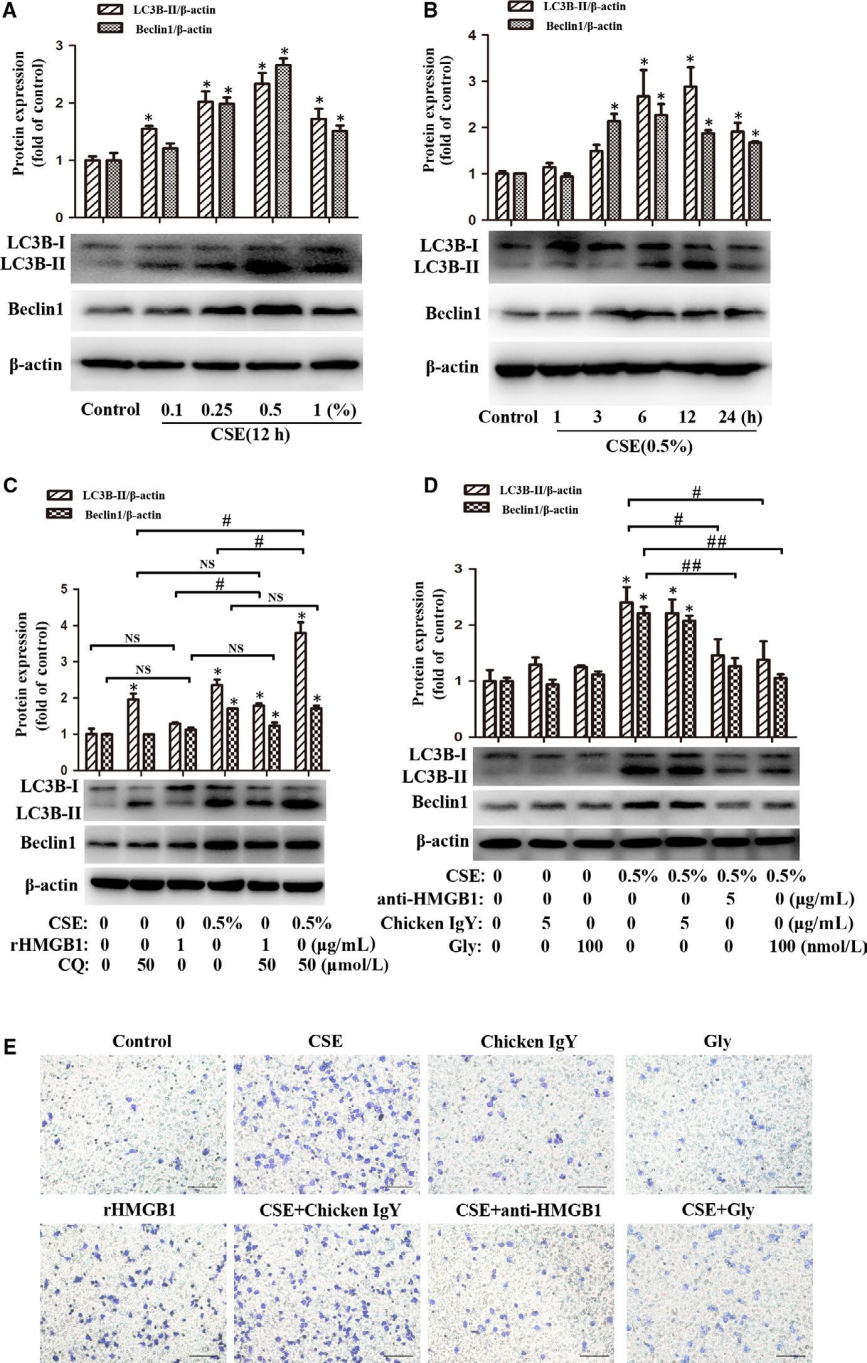
Blockade of HMGB1 attenuated CSE‐induced autophagy and migration of lung macrophages. A, After MH‐S cells were treated with CSE (0, 0.1%, 0.25%, 0.5% or 1%) for 12 h, LC3B‐I/ II and Beclin1 were detected with Western blot. B, After MH‐S cells were treated with 0.5% CSE for 1, 3, 6, 12 or 24 h, LC3B‐I/ II and Beclin1 were detected with Western blot. C, After MH‐S cells were pre‐treated with CQ (50 μmol/L) for 2 h, CSE (0.5%) or rHMGB1 (1 μg/mL) were then added to stimulation for 12 h. D, MH‐S cells were pre‐treated with chicken IgY (5 μg/mL), anti‐HMGB1 (5 μg/mL) or Gly (100 nmol/L) for 90 min before 0.5% CSE incubated for 12 h. E, MH‐S cells were pre‐treated with chicken IgY (5 μg/mL), anti‐ HMGB1 (5 μg/mL) or Gly (100 nmol/L) for 90 min before 0.5% CSE or rHMGB1 (1 μg/mL) alone incubated for 24 h. Migration assay was assessed. Bar: 100 μm.*P < .05 vs Control group. ^#^ *P* < .05. ^##^ *P* < .05. NS, no significant. Values are mean ± SEM of three independent experiments

Previous studies demonstrated that CSE induced autophagy and migration of macrophages.^7^, ^10^, ^11^, ^26^ Autophagy was measured by detecting the biochemical marker LC3B‐II which was modulated through Beclin1.^7^, ^11^ MH‐S cells were stimulated as described above by various concentrations of CSE (0, 0.1%, 0.25%, 0.5% or 1%) for 12 hours (Figure 5A) or by CSE (0.5%) for indicated time (1, 3, 6, 12, or 24 hours) (Figure 5B). Indeed, CSE induced up‐regulation of LC3B‐II and Beclin1 in a concentration‐dependent and time‐dependent manner, suggesting the activation of autophagy, while rHMGB1 also induced increase but not statistic significant of LC3B‐II (*P* = .239) and Beclin1 (*P* = .124) expression in MH‐S cells (Figure 5C). CQ, an autophagy inhibitor via suppressing autophagosome‐lysosome fusion, significantly enhanced the increase of LC3B‐II (*P* = .0001, *P = *.048, respectively) but not Beclin1 (*P* = .955, *P = *.138, respectively) in CSE or rHMGB1 treated MH‐S cells, indicating induction of autophagy by CSE and rHMGB1.

Since CSE induced up‐regulation of HMGB1 in macrophages, we asked whether HMGB1 was involved in cellular autophagy. Neutralizing antibody (anti‐HMGB1) and HMGB1 inhibitor (Gly) were respectively added to MH‐S cell cultures before CSE stimulation. Interestingly, blockade of HMGB1 significantly attenuated the increase of LC3B‐II and Beclin1 (Figure 5D) in CSE‐stimulated MH‐S cells. Taken together, these results indicated the involvement of HMGB1 in CSE‐induced autophagy through modulating Beclin1.

### 3.7 Blockade of HMGB1 attenuated CSE‐induced migration of lung macrophages

Macrophages were increased in lung tissues and BALF of COPD patients, and CSE was able to trigger recruitment of macrophages.^26^, ^27^ As expected, here, we found that CSE induced migration of MH‐S cells, while blockade of HMGB1 by anti‐HMGB1 or Gly attenuated CSE‐induced cell migration (Figure 5E). Our result also showed that rHMGB1 alone induced migration of MH‐S cells (Figure 5E).

### 3.8 Blockade of HMGB1 attenuated CSE‐induced activation of NF‐κB signalling in lung macrophages

**Figure 6:**
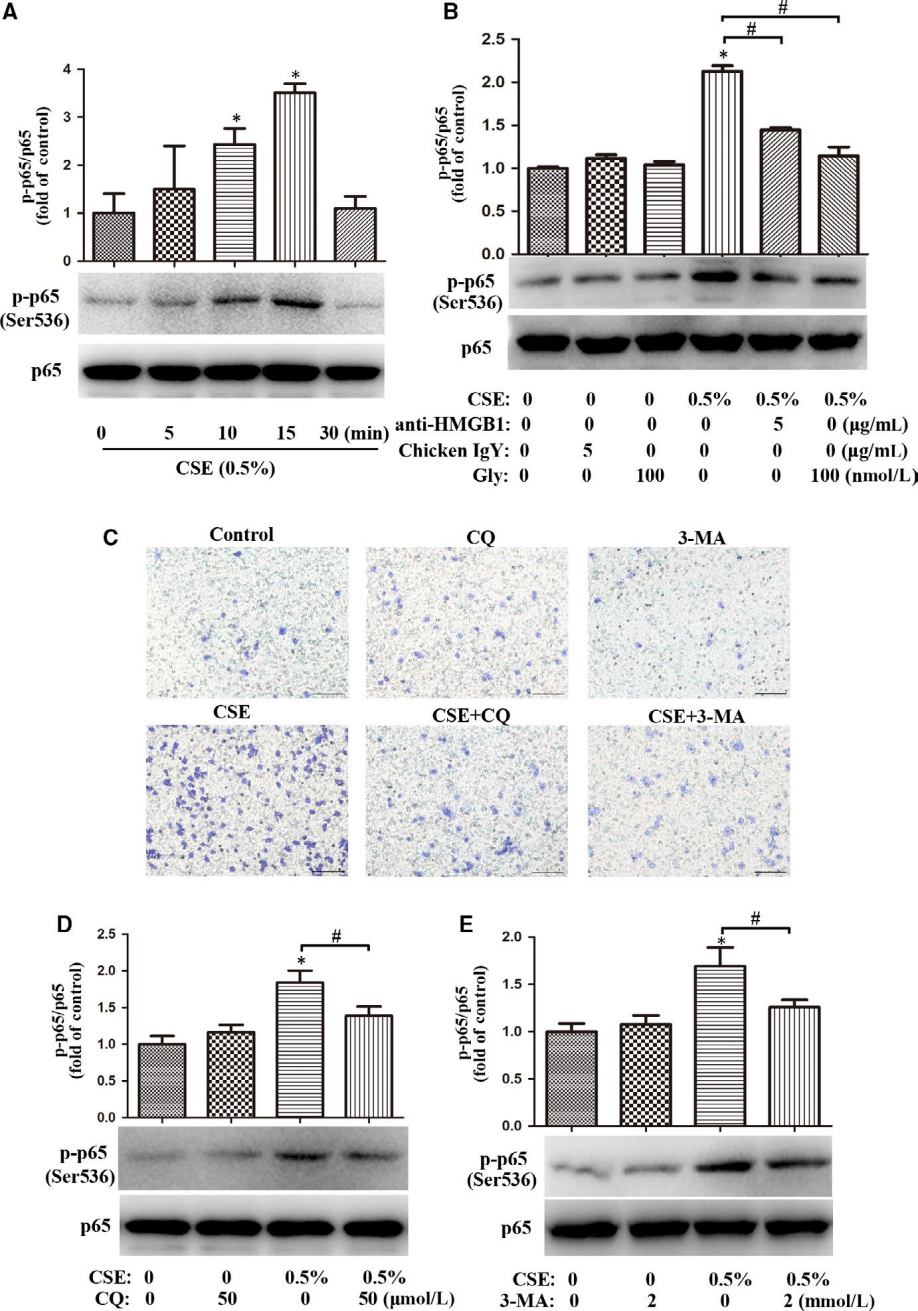
Inhibition of autophagy abrogated the migration and NF‐κB activation in CSE‐treated lung macrophages. A, After MH‐S cells were treated with 0.5% CSE for 0, 5, 10, 15 or 30 min, the level of p‐p65 (Ser536) was assessed by Western blot. B, MH‐S cells were pre‐treated with chicken IgY (5 μg/mL), anti‐HMGB1 (5 μg/mL) or Gly (100 nmol/L) for 90 min, followed by 0.5% CSE incubation for 15 min. The level of p65 phosphorylation was assessed by Western blot. C, MH‐S cells were pre‐treated with CQ (50 μmol/L) or 3‐MA (2 mmol/L) for 2 h, followed by 0.5% CSE incubation for 24 h. Migration assay was assessed. Bar: 100 μm. (D‐E) MH‐S cells were pre‐treated with CQ (50 μmol/L) or 3‐MA (2 mmol/L) for 2 h, followed by 0.5% CSE incubation for 15 min. The level of p‐p65 (Ser536) was assessed by Western blot. *P < .05 vs Control group. ^#^ *P* < .05. Values are mean ± SEM of three independent experiments

NF‐κB signalling activation, by promoting transcription of pro‐inflammatory cytokines, plays critical roles in the pathogenesis of COPD.^28^, ^29^ Pattern‐recognition molecules promoted NF‐κB p65 translocation to participate in the regulation of macrophage function.^30^ Thus, we asked whether HMGB1 mediated NF‐κB signalling induced by CSE stimulation in lung macrophages. Our Western blot results showed that CSE induced robust p65 phosphorylation at 15 minutes (Figure 6A), and blockade of HMGB1 by anti‐HMGB1 or Gly attenuated CSE‐induced p65 phosphorylation at 15 minutes in MH‐S cells (Figure 6B), indicating that HMGB1 mediated CSE‐induced activation of NF‐κB signalling.

### 3.9 Inhibition of autophagy abrogated migration and NF‐κB activation of CSE‐treated lung macrophages

Previous studies found that autophagy mediated regulation of macrophage recruitment and NF‐κB signalling pathway.^5^, ^31^ We asked whether inhibition of autophagy by CQ or 3‐MA had any effect on CSE‐induced migration and NF‐κB activation of lung macrophages. As expected, CQ (50 μmol/L) or 3‐MA (2 mmol/L) abrogated CSE‐induced migration of MH‐S cells (Figure 6C) and attenuated CSE‐induced p65 phosphorylation at 15 minutes in these cells (Figure 6D‐E).

## 4 DISCUSSION

In the present study, we demonstrated up‐regulation, nucleocytoplasmic translocation and release of HMGB1 in lung macrophages from COPD patients and CS‐exposed mice. We further confirmed in vitro studies that CSE induced, through nAChR, HMGB1 up‐regulation, translocation and release, which was involved in CSE‐induced autophagy, migration and NF‐κB activation of lung macrophages. Besides, we also found that inhibition of autophagy abrogated CSE‐induced migration and NF‐κB activation of lung macrophages.

High‐mobility group box 1 is a multifunctional protein, widely expressed in the nuclei of macrophages, epithelial and endothelial cells.^12^, ^13^ Our results also revealed that HMGB1 was also highly expressed in macrophages of lung tissue and other pulmonary structure cells. On one hand, HMGB1 can stabilize chromatin structure and regulate gene transcription in the nucleus. On the other hand, HMGB1 can be exported from the nucleus to the cytoplasm and released to extracellular milieu, exerting pro‐inflammatory effects, which have been involved in various lung inflammatory diseases.^12^, ^13^, ^32^, ^33^ Recent studies have implicated HMGB1 in the pathogenesis of COPD.^19^, ^20^, ^34^ CS impaired lung function and increased HMGB1 expression in a chronic (60 days) CS‐exposure mouse model.^35^ A previous study also revealed that CS induced HMGB1 translocation and release from lung epithelial cells, and HMGB1 further induced an inflammatory response through TLR4/MyD88‐dependent signalling in an acute (3 days) CS‐exposure model.^21^ Released HMGB1 also induced cytokine release through TLR4 receptors in macrophages.^36^ However, HMGB1 intracellular localization and release had not been described in COPD patients and long‐term (24 weeks) CS‐exposure models. Here, we found marked expression of HMGB1 in lung tissues and peripheral blood from COPD patients, along with predominantly cytoplasmic exporting of this molecule from the nucleus in lung macrophages, indicating up‐regulated expression and nucleocytoplasmic translocation of HMGB1 in lung macrophages of COPD. These findings were recapitulated in lung macrophages from a well‐established long‐term CS‐exposure mouse model. Subsequent experiments with MH‐S cells also confirmed that CSE induced expression, nucleocytoplasmic translocation and release of HMGB1. Considering that nAChR, the receptor of nicotine (a major component of cigarette), is highly expressed in macrophages and plays an essential role in immunomodulation of macrophages,^26^, ^37^ we further demonstrated that MEC, a competitive inhibitor to nAChR, reversed CSE‐induced HMGB1 expression in nuclear protein, cytoplasmic protein, whole protein and extracellular protein, suggesting that nAChR was involved in CSE‐induced nucleocytoplasmic translocation and release of HMGB1 in lung macrophages.

Lung macrophages play a critical role in maintaining of lung homeostasis and regulation of innate and acquired immunity, which is closely related to the process of COPD.^2^, ^3^, ^27^, ^38^ Autophagy is a fundamental intracellular process responsible for regulation of lung macrophage. Cigarette smoking induces autophagy of lung macrophages, and excessive autophagy aggravates lung macrophage dysfunction.^5^, ^6^, ^26^, ^39^ Dysfunction of lung macrophages results in down‐regulation of phagocytosis and bacterial clearance on one hand, and promotes release of inflammatory mediators and proteases on the other. These consequences contribute to airway inflammation and alveolar destruction, major characteristics of COPD and its exacerbation.^2^, ^3^, ^4^, ^27^ Recent studies demonstrated that HMGB1 promoted autophagy in lung ischaemia‐reperfusion/injury‐triggered macrophages, facilitating the inflammation response.^22^, ^23^ Consistent with these studies, we found that blockade of HMGB1 with anti‐HMGB1 and Gly attenuated CSE‐induced increase of LC3B‐II, suggesting the involvement of HMGB1 in CSE‐induced autophagy. We further confirmed this finding by inhibition of autophagy with CQ. Taken together, these data indicate that HMGB1 is a major culprit for CSE‐induced autophagy of macrophages, suggesting that it may be a potential target for therapeutic intervention.

CS triggers recruitment of blood monocytes into the lung, accounting for the increase of macrophages in pulmonary alveoli and parenchyma of patients with COPD.^26^, ^27^ Meanwhile, the migration ability of macrophages and migratory chemokines such as CXCR3 and CCR5 are increased in the process of COPD.^26^, ^40^ Several studies revealed that HMGB1 activated the RAGE/NF‐κB pathway and promoted cell migration, an event accompanying the recruitment of monocytes.^41^, ^42^ Consistent with these studies, our results showed that CSE induced migration of lung macrophages, and blockade of HMGB1 with anti‐HMGB1 or Gly significantly attenuated CSE‐induced migration of these cells. Exogenous rHMGB1 also induced migration of lung macrophages but its ability to induce migration was weaker than CSE, suggesting that CSE induced migration of lung macrophages partly through HMGB1. Interestingly, we also found that CQ and 3‐MA abrogated CSE‐induced migration of lung macrophages through blocking autophagy, indicating that CSE‐induced HMGB1 translocation and release contribute to macrophage migration via induction of autophagy.

NF‐κB signalling is the critical signal molecule in inflammation of airways, contributing to development of COPD.^28^, ^43^ A previous study reported that HMGB1 mediated aspergillus fumigatus‐induced inflammatory response in macrophages of COPD mice via activating MyD88/NF‐κB and syk/PI3K signalling.^44^ In the current study, we showed that CSE‐induced NF‐κB activation in lung macrophages was mediated through HMGB1, and more intriguingly, CQ and 3‐MA abrogated CSE‐induced NF‐κB activation in lung macrophages through blocking autophagy, indicating that CSE‐induced HMGB1 translocation and release contribute to NF‐κB activation through inducing autophagy in lung macrophages, thereby promoting the transcription of downstream inflammatory cytokines.

## 5 CONCLUSION

**Figure 7:**
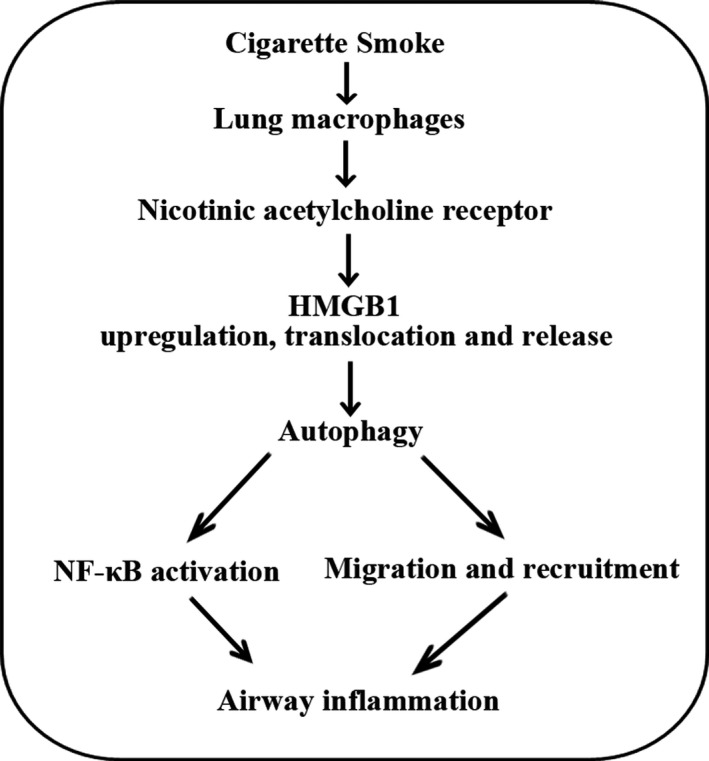
Diagram summarizing the mechanism by which CS‐induced HMGB1 translocation and release contribute to migration and NF‐κB activation by inducing autophagy in lung macrophages

In summary, we demonstrated, for the first time to our knowledge, that CS induced HMGB1 translocation and release in lung macrophages through nAChR, and released HMGB1 contributed to CSE‐induced NF‐κB activation and migration of lung microphages through inducing autophagy (Figure 7). Our study provides further evidence that HMGB1 is an important regulator of CS‐induced airway inflammatory response of lung macrophages in the pathogenesis of COPD as well as a potentially new target for intervention.

## CONFLICT OF INTEREST

The authors confirm that there are no conflicts of interest.

## AUTHORS' CONTRIBUTIONS

Le YQ, Wang YH and Sun YC designed the experiments; Le YQ, Zhou L, Xiong J, Tian JY, Yang X and Gai XY performed the experiments; Le YQ, Wang YH and Zhou L analysed the data; Le YQ, Wang YH and Sun YC wrote the paper.

## Data Availability

The data that support the findings of this study are available on request from the corresponding author.

## References

[1] Global Strategy for the Diagnosis, Management and Prevention of COPD . Global Initiative for Chronic Obstructive Lung Disease (GOLD).2019; http://www.goldcopd.org

[2] Byrne AJ , Mathie SA , Gregory LG , et al. Pulmonary macrophages: key players in the innate defence of the airways. Thorax. 2015;70:1189‐1196. 26286722 10.1136/thoraxjnl-2015-207020

[3] Kapellos TS , Bassler K , Aschenbrenner AC , et al. Dysregulated functions of lung macrophage populations in COPD. J Immunol Res. 2018;2018:2349045. 29670919 10.1155/2018/2349045 PMC5835245

[4] Yamasaki K , Eeden SFV . Lung macrophage phenotypes and functional responses: role in the pathogenesis of COPD. Int J Mol Sci. 2018: 19(2):582. 10.3390/ijms19020582 PMC5855804 29462886

[5] Chen P , Cescon M , Bonaldo P . Autophagy‐mediated regulation of macrophages and its applications for cancer. Autophagy. 2014;10:192‐200. 24300480 10.4161/auto.26927 PMC5396097

[6] Sarkar A , Tindle C , Pranadinata RF , et al. ELMO1 regulates autophagy induction and bacterial clearance during enteric infection. J Infect Dis. 2017;216:1655‐1666. 29029244 10.1093/infdis/jix528 PMC5853658

[7] Kim HP , Wang X , Chen ZH , et al. Autophagic proteins regulate cigarette smoke‐induced apoptosis: protective role of heme oxygenase‐1. Autophagy. 2008;4:887‐895. 18769149 10.4161/auto.6767

[8] Chen ZH , Kim HP , Sciurba FC , et al. Egr‐1 regulates autophagy in cigarette smoke‐induced chronic obstructive pulmonary disease. PLoS ONE. 2008;3:e3316. 18830406 10.1371/journal.pone.0003316 PMC2552992

[9] Ghavami S , Shojaei S , Yeganeh B , et al. Autophagy and apoptosis dysfunction in neurodegenerative disorders. Prog Neurogibol. 2014;112:24‐49. 10.1016/j.pneurobio.2013.10.004 24211851

[10] Hwang JW , Chung S , Sundar IK , et al. Cigarette smoke‐induced autophagy is regulated by SIRT1‐PARP‐1‐dependent mechanism: implication in pathogenesis of COPD. Arch Biochem Biophys. 2010;500:203‐209. 20493163 10.1016/j.abb.2010.05.013 PMC2904411

[11] Li L , Zhang M , Zhang L , et al. Klotho regulates cigarette smoke‐induced autophagy: implication in pathogenesis of COPD. Lung. 2017;195:295‐301. 28349330 10.1007/s00408-017-9997-1

[12] Ding J , Cui X , Liu Q . Emerging role of HMGB1 in lung diseases: friend or foe. J Cell Mol Med. 2017;21:1046‐1057. 28039939 10.1111/jcmm.13048 PMC5431121

[13] Musumeci D , Roviello GN , Montesarchio D . An overview on HMGB1 inhibitors as potential therapeutic agents in HMGB1‐related pathologies. Pharmacol Ther. 2014;141:347‐357. 24220159 10.1016/j.pharmthera.2013.11.001

[14] Zhang L , Liu M , Jiang H , et al. Extracellular high‐mobility group box 1 mediates pressure overload‐induced cardiac hypertrophy and heart failure. J Cell Mol Med. 2016;20:459‐470. 26647902 10.1111/jcmm.12743 PMC4759479

[15] Harris HE , Andersson U , Pisetsky DS . HMGB1: a multifunctional alarmin driving autoimmune and inflammatory disease. Nat Rev Rheumatol. 2012;8:195‐202. 22293756 10.1038/nrrheum.2011.222

[16] Yang L , Xie M , Yang M , et al. PKM2 regulates the Warburg effect and promotes HMGB1 release in sepsis. Nat Commun. 2014;5:4436. 25019241 10.1038/ncomms5436 PMC4104986

[17] Wang Y , Le Y , Zhao W , et al. Short thymic stromal lymphopoietin attenuates toluene diisocyanate‐induced airway inflammation and inhibits high mobility group Box 1‐receptor for advanced glycation end products and long thymic stromal lymphopoietin expression. Toxicol Sci. 2017;157:276‐290. 28329851 10.1093/toxsci/kfx043

[18] Lundback P , Stridh P , Klevenvall L , et al. Characterization of the inflammatory properties of actively released HMGB1 in juvenile idiopathic arthritis. Antioxid Redox Signal. 2016;24:605‐619. 25532033 10.1089/ars.2014.6039 PMC4841912

[19] Ko HK , Hsu WH , Hsieh CC , et al. High expression of high‐mobility group box 1 in the blood and lungs is associated with the development of chronic obstructive pulmonary disease in smokers. Respirology. 2014;19:253‐261. 24372740 10.1111/resp.12209

[20] Hou C , Zhao H , Liu L , et al. High mobility group protein B1 (HMGB1) in Asthma: comparison of patients with chronic obstructive pulmonary disease and healthy controls. Mol Med. 2011;17:807‐815. 21380479 10.2119/molmed.2010.00173 PMC3146613

[21] Cheng Y , Wang D , Wang B , et al. HMGB1 translocation and release mediate cigarette smoke‐induced pulmonary inflammation in mice through a TLR4/MyD88‐dependent signaling pathway. Mol Biol Cell. 2017;28:201‐209. 27807045 10.1091/mbc.E16-02-0126 PMC5221624

[22] Liu X , Cao H , Li J , et al. Autophagy induced by DAMPs facilitates the inflammation response in lungs undergoing ischemia‐reperfusion injury through promoting TRAF6 ubiquitination. Cell Death Differ. 2017;24:683‐693. 28157209 10.1038/cdd.2017.1 PMC5384028

[23] Yanai H , Matsuda A , An J , et al. Conditional ablation of HMGB1 in mice reveals its protective function against endotoxemia and bacterial infection. Proc Natl Acad Sci U S A. 2013;110:20699‐20704. 24302768 10.1073/pnas.1320808110 PMC3870753

[24] Zhou L , Le Y , Tian J , et al. Cigarette smoke‐induced RANKL expression enhances MMP‐9 production by alveolar macrophages. Int J Chron Obstruct Pulmon Dis. 2019;14:81‐91. 30587964 10.2147/COPD.S190023 PMC6304243

[25] Ferhani N , Letuve S , Kozhich A , et al. Expression of high‐mobility group box 1 and of receptor for advanced glycation end products in chronic obstructive pulmonary disease. Am J Respir Crit Care Med. 2010;181:917‐927. 20133931 10.1164/rccm.200903-0340OC

[26] Bai X , Stitzel JA , Bai A , et al. Nicotine impairs macrophage control of mycobacterium tuberculosis. Am J Respir Cell Mol Biol. 2017;57:324‐333. 28398760 10.1165/rcmb.2016-0270OC PMC5625222

[27] Vlahos R , Bozinovski S . Role of alveolar macrophages in chronic obstructive pulmonary disease. Front Immunol. 2014;5:435. 25309536 10.3389/fimmu.2014.00435 PMC4160089

[28] Schuliga M . NF‐kappaB signaling in chronic inflammatory airway disease. Biomolecules. 2015;5:1266‐1283. 26131974 10.3390/biom5031266 PMC4598751

[29] Sun X , Feng X , Zheng D , et al. Ergosterol attenuates cigarette smoke extract‐induced COPD by modulating inflammation, oxidative stress and apoptosis in vitro and in vivo. Clin Sci (Lond). 2019;133:1523‐1536. 31270147 10.1042/CS20190331

[30] Liu YS , Wang LF , Cheng XS , et al. The pattern‐recognition molecule mindin binds integrin Mac‐1 to promote macrophage phagocytosis via Syk activation and NF‐kappaB p65 translocation. J Cell Mol Med. 2019;23:3402‐3416. 30869196 10.1111/jcmm.14236 PMC6484411

[31] Pawlowska E , Szczepanska J , Wisniewski K , et al. NF‐κB‐mediated inflammation in the pathogenesis of intracranial aneurysm and subarachnoid hemorrhage. does autophagy play a role? Int J Mol Sci. 2018;19(4):1245. 10.3390/ijms19041245 PMC5979412 29671828

[32] Seidu RA , Wu M , Su Z , et al. Paradoxical role of high mobility group box 1 in glioma: a suppressor or a promoter? Oncol Rev. 2017;11:325. 28382190 10.4081/oncol.2017.325 PMC5364998

[33] Yang PS , Kim DH , Lee YJ , et al. Glycyrrhizin, inhibitor of high mobility group box‐1, attenuates monocrotaline‐induced pulmonary hypertension and vascular remodeling in rats. Respir Res. 2014;15:148. 25420924 10.1186/s12931-014-0148-4 PMC4248446

[34] Gangemi S , Casciaro M , Trapani G , et al. Association between HMGB1 and COPD: a systematic review. Mediators Inflamm. 2015;2015:164913. 26798204 10.1155/2015/164913 PMC4698778

[35] Bezerra FS , Valenca SS , Pires KM , et al. Long‐term exposure to cigarette smoke impairs lung function and increases HMGB‐1 expression in mice. Respir Physiol Neurobiol. 2011;177:120‐126. 21457800 10.1016/j.resp.2011.03.023

[36] Yang H , Hreggvidsdottir HS , Palmblad K , et al. A critical cysteine is required for HMGB1 binding to Toll‐like receptor 4 and activation of macrophage cytokine release. Proc Natl Acad Sci U S A. 2010;107:11942‐11947. 20547845 10.1073/pnas.1003893107 PMC2900689

[37] Galvis G , Lips KS , Kummer W . Expression of nicotinic acetylcholine receptors on murine alveolar macrophages. J Mol Neurosci. 2006;30:107‐108. 17192650 10.1385/JMN:30:1:107

[38] Zabini D , Crnkovic S , Xu H , et al. High‐mobility group box‐1 induces vascular remodelling processes via c‐Jun activation. J Cell Mol Med. 2015;19:1151‐1161. 25726846 10.1111/jcmm.12519 PMC4420616

[39] Qian Q , Cao X , Wang B , et al. TNF‐alpha‐TNFR signal pathway inhibits autophagy and promotes apoptosis of alveolar macrophages in coal worker's pneumoconiosis. J Cell Physiol. 2019;234:5953‐5963. 30467847 10.1002/jcp.27061

[40] Costa C , Traves SL , Tudhope SJ , et al. Enhanced monocyte migration to CXCR3 and CCR5 chemokines in COPD. Eur Respir J. 2016;47:1093‐1102. 26965295 10.1183/13993003.01642-2015

[41] Vogel S , Rath D , Borst O , et al. Platelet‐derived high‐mobility group box 1 promotes recruitment and suppresses apoptosis of monocytes. Biochem Biophys Res Commun. 2016;478:143‐148. 27449608 10.1016/j.bbrc.2016.07.078 PMC4976924

[42] Gerhardt T , Ley K . Monocyte trafficking across the vessel wall. Cardiovasc Res. 2015;107:321‐330. 25990461 10.1093/cvr/cvv147 PMC4592323

[43] Yuan J , Liu R , Ma Y , et al. Curcumin attenuates airway inflammation and airway remolding by inhibiting NF‐kappaB signaling and COX‐2 in cigarette smoke‐induced COPD mice. Inflammation. 2018;41:1804‐1814. 29961171 10.1007/s10753-018-0823-6

[44] Zhang P , Xin X , Fang L , et al. HMGB1 mediates Aspergillus fumigatus‐induced inflammatory response in alveolar macrophages of COPD mice via activating MyD88/NF‐kappaB and syk/PI3K signalings. Int Immunopharmacol. 2017;53:125‐132. 29078091 10.1016/j.intimp.2017.10.007

